# Parallel Recordings of Transmembrane hERG Channel Currents Based on Solvent-Free Lipid Bilayer Microarray

**DOI:** 10.3390/mi12010098

**Published:** 2021-01-19

**Authors:** Ryusuke Miyata, Daisuke Tadaki, Daichi Yamaura, Shun Araki, Madoka Sato, Maki Komiya, Teng Ma, Hideaki Yamamoto, Michio Niwano, Ayumi Hirano-Iwata

**Affiliations:** 1 Laboratory for Nanoelectronics and Spintronics, Research Institute of Electrical Communication, Tohoku University, 2-1-1 Katahira, Aoba-ku, Sendai 980-8577, Japan; ryusuke.miyata.s2@dc.tohoku.ac.jp (R.M.); daisuke.tadaki@tohoku.ac.jp (D.T.); daichi.yamaura.t5@dc.tohoku.ac.jp (D.Y.); arakishun@ecei.tohoku.ac.jp (S.A.); madoka.sato.p6@dc.tohoku.ac.jp (M.S.); maki.komiya.c7@tohoku.ac.jp (M.K.); hideaki.yamamoto.e3@tohoku.ac.jp (H.Y.); 2 Advanced Institute for Materials Research, Tohoku University, 2-1-1 Katahira, Aoba-ku, Sendai 980-8577, Japan; teng.ma@tohoku.ac.jp; 3 Core Research Cluster for Materials Science, Tohoku University, 2-1-1 Katahira, Aoba-ku, Sendai 980-8577, Japan; 4 Kansei Fukushi Research Institute, Tohoku Fukushi University, 6-149-1 Kunimi-ga-oka, Aoba-ku, Sendai 989-3201, Japan; niwano@riec.tohoku.ac.jp

**Keywords:** bilayer lipid membrane (BLM), ion channel, human *ether-a-go-go-*related gene (hERG) channel, microarray

## Abstract

The reconstitution of ion-channel proteins in artificially formed bilayer lipid membranes (BLMs) forms a well-defined system for the functional analysis of ion channels and screening of the effects of drugs that act on these proteins. To improve the efficiency of the BLM reconstitution system, we report on a microarray of stable solvent-free BLMs formed in microfabricated silicon (Si) chips, where micro-apertures with well-defined nano- and micro-tapered edges were fabricated. Sixteen micro-wells were manufactured in a chamber made of Teflon^®^, and the Si chips were individually embedded in the respective wells as a recording site. Typically, 11 to 16 BLMs were simultaneously formed with an average BLM number of 13.1, which corresponded to a formation probability of 82%. Parallel recordings of ion-channel activities from multiple BLMs were successfully demonstrated using the human *ether-a-go-go-*related gene (hERG) potassium channel, of which the relation to arrhythmic side effects following drug treatment is well recognized.

## 1. Introduction

The cell membrane is composed of a bilayer lipid membrane (BLM), a self-assembled structure of phospholipid membrane molecules, and membrane proteins embedded in the BLM [1]. Among various membrane proteins, ion-channel proteins function as gated pores that permit ion fluxes across membranes that have a considerably high resistance (>1 GΩ). Because ion channels play an essential role in transmembrane signaling, they are attractive as a major target for drug design [2,3,4,5]. Electrical recording of transmembrane ion-channel currents is one of the most efficient methods for characterizing the channel functions and screening the effects of drugs acting on ion-channel proteins [6,7]. Fluorometric measurement of transmembrane ion fluxes using liposomes [8] or BLMs suspended on micro- and nano-cavity arrays [9,10] is also useful for high-throughput recordings of channel functions. However, only electrical recording can control and clamp the transmembrane voltage, which is necessary for the functional analysis of voltage-gated ion channels. Although the patch-clamping method has been the gold standard for the analysis of drug actions on ion channels including voltage-gated ion channels [11,12,13], the observed currents are prone to be influenced by the conditions of the target cells [6], and a time-consuming process is necessary to prepare well-conditioned cell samples. Meanwhile, artificial cell membrane systems based on BLMs with reconstituted ion channels have garnered attention as a complementary method to patch-clamping [14,15,16,17,18]. The BLM systems provide a significant advantage, and the composition of the system, including the buffer solution and membrane components, can be easily and precisely controlled. However, the BLM reconstitution system suffers from low experimental efficiency and throughput owing to the instability of BLMs.

In BLM reconstitution systems, free-standing BLMs are commonly suspended in micro-apertures fabricated in plastic films [17,18,19], glass pipettes [20,21,22], and microstructured silicon nitride (Si_3_N_4_)/silicon (Si) septa [15,23,24]. Although decreasing the size of the aperture significantly improves the stability of BLMs, incorporating ion channels into smaller BLMs becomes more difficult [18,25]. Among numerous attempts to overcome this trade-off, we focused on the shape of the aperture edge and reported on the fabrication of micro-apertures with smoothly tapered edges in Si_3_N_4_/Si chips [26,27,28]. Si_3_N_4_ provides various advantages, such as compatibilities with well-established fabrication technology, chemical stability, and high mechanical robustness [29]. By utilizing these properties of Si_3_N_4_, mechanically stable solvent-free BLMs were reproducibly formed in micro-apertures with a micro-tapered structure, in addition to a nano-tapered edge, and were applied to electrical recordings of channel activities of the human *ether-a-go-go-*related gene (hERG) channel, a cardiac voltage-gated potassium channel [28,30,31].

Non-volatile organic solvents have been frequently used to stabilize BLMs [1,15,16,17,18,19,20,21,22,23]. The solvent seals the gap between the nanometer-thick BLMs and aperture walls, and it allows lipid molecules to align in an appropriate orientation. However, the presence of organic solvents seems problematic when we consider the applications of BLM systems to drug-screening platforms. This is because hydrophobic drugs are partitioned into organic solvents. This may cause a difficulty in controlling the drug concentration in the water phase [25,32]. Therefore, it is highly important to construct solvent-free BLM systems for efficient screening of drug effects on ion-channel proteins.

For constructing high-throughput screening platforms for transmembrane ion channels, there is a growing interest in the development of multiple BLM array systems [33]. Several BLM array platforms have been reported, such as microfluidic arrays [34,35], droplet interface bilayer (DIB) arrays [36,37], and microcavity BLM arrays [38,39]. However, these approaches are commonly based on BLMs prepared from lipids dissolved in non-volatile organic solvents. Solvent-free BLM arrays can be formed by rupturing giant unilamellar vesicles over micro- and nano-cavity arrays [9,10], but simultaneous electrical measurements from respective BLMs have not been reported. Conversely, we previously reported a prototype Si_3_N_4_/Si chip array, wherein stable BLMs were simultaneously formed in microfabricated tapered apertures [40]. Nine micro-wells were manufactured in a chamber made of Teflon^®^, and the Si_3_N_4_/Si chips were individually embedded in the respective wells to function as recording sites. This prototype BLM array required *n-*hexadecane for the formation of stable BLMs; therefore, we recently optimized the tapered-edge structures of micro-apertures, which enabled reproducible formation of stable solvent-free BLMs [28]. In the present study, we combined the array system with solvent-free BLMs and extended the number of recording sites from 9 to 16. The structures of the Teflon^®^ chamber were also optimized to decrease the required amounts of protein samples, which was necessary for the application to ion channels of pharmaceutical interest. As an illustrative example, we attempted parallel recordings of the channel activities of the hERG potassium channel at the single-molecule level.

## 2. Materials and Methods

### 2.1. Reagents and Cell Lines

Chloroform solutions of L-α-phosphatidylcholine (Egg-PC) and L-α-phosphatidylethanolamine (Egg-PE) were purchased from Avanti Polar Lipids (Alabaster, AL, USA). Cholesterol (Chol) was obtained from Wako Pure Chemicals (Osaka, Japan) and recrystallized three times from methanol. Human embryonic kidney (HEK) 293 cell lines expressing hERG channels were purchased from Anaxon AG (Bern, Switzerland) and cultured in a 37 °C incubator with 5% CO_2_, according to the manufacturer’s protocol. The cell lines were maintained in Dulbecco’s modified Eagle’s medium/GlutaMAX medium supplemented with 10% fetal bovine serum and 1% penicillin/streptomycin under an antibiotic pressure of 100 μg/mL geneticin (Gibco, Waltham, MA, USA). The hERG channels were extracted from the HEK 293 cell lines as membrane fractions, according to the procedures described by Hirano-Iwata et al. [41]. The final membrane pellets were resuspended in 120 mM KCl solution containing 10 mM HEPES/KOH (pH 7.2) (KCl buffer) to form proteoliposomes. E-4031, a specific blocker of hERG channels [42,43], was obtained from Wako Pure Chemicals. (3-Cyanopropyl)dimethylchlorosilane (CPDS) and acetonitrile (for HPLC) were purchased from Gelest (Morrisville, PA, USA) and Wako Pure Chemicals, respectively.

### 2.2. Preparation of Si Chips

**Figure 1:**
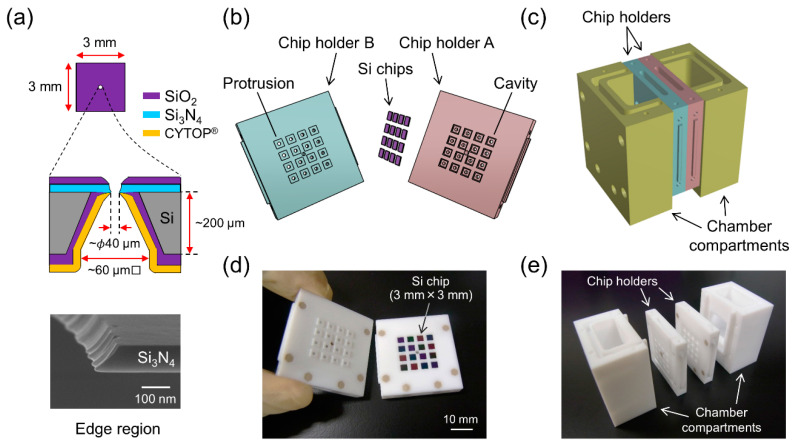
(**a**) Schematic drawing of single Si chip. (upper) Top view from the Si_3_N_4_ side. (middle) Cross-sectional side view. (bottom) Field emission scanning electron microscopy (FE-SEM) image around the edge region of the micro-aperture. Tilt angle: 65°. (**b**) Schematic drawing of chip holders and Si chips. (**c**) Schematic drawing of the chamber system that is held together with polyetheretherketone (PEEK) screws. (**d**) Photograph of chip holders A and B. Sixteen Si chips are placed in chip holder A. (**e**) Photograph of the components used to assemble the system.

Si chips with a size of 3 mm × 3 mm having micro-apertures with a diameter of ~40 μm (Figure 1a) were fabricated according to the procedures described by Tadaki et al. [28]. Since the size of the Si chips in the present study was smaller than that in the previous study (5 mm × 5 mm), the process of coating CYTOP^®^ (insulating polymer) was modified. In the previous study, a CYTOP^®^ coating was applied to each 5 mm × 5 mm Si chip, while in the present study, the same coating was applied to a 20 mm × 20 mm Si substrate that contained 25 chips with a size of 3 mm × 3 mm. The fabricated Si chips were cleaned by rinsing in chloroform, ethanol, acetone, and acetonitrile, followed by ashing using an air plasma (PM100, Yamato Scientific, Tokyo, Japan). After rinsing again with acetonitrile, the substrates were immersed in a 2% (*v*/*v*) solution of CPDS dissolved in acetonitrile for 1–2 h at room temperature in a nitrogen-filled environment.

### 2.3. Assembling Microarray System

**Figure 2:**
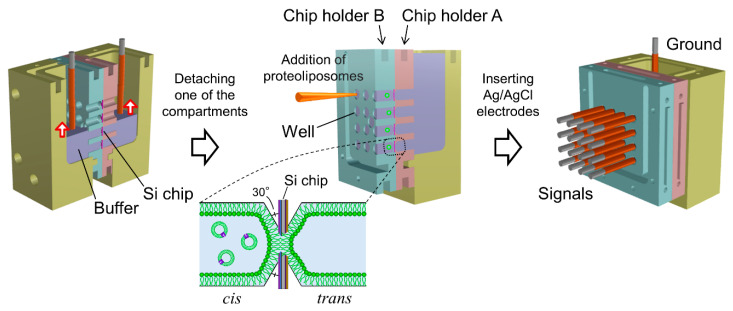
Schematic images of procedures for simultaneous formation of bilayer lipid membranes (BLMs) and rearrangement for multiarray measurements.

A Teflon^®^ chamber system for placing 16 fabricated Si chips was manufactured by IVY-MODEL (Saitama, Japan). As shown in Figure 1, the chamber consists of two chip holders (A and B) and two compartments for the buffer solution. Chip holder A has 16 cavities for placing the 3 mm × 3 mm Si chips. Chip holder B has the same number of protrusions to hold the chips. Holder B also contains 16 small wells on the other side of the protrusions (Figure 2). Each well functions as a solution reservoir with a volume of approximately 40 μL. There were 16 openings of ϕ1 mm in both the cavities of holder A and the wells of holder B. The two holders (A and B) were docked together and then attached to the two compartments. All the holders and compartments were assembled with polyetheretherketone (PEEK) screws. When the compartments were filled with KCl buffer, the Si chips were in contact with the KCl buffer on both sides through the openings.

### 2.4. BLM Formation

Sixteen BLMs were simultaneously formed in the micro-apertures via the folding method, in which two lipid monolayers spread at an air/buffer interface were hybridized in a small aperture by gradually increasing the buffer level [14]. A 1.4 mL portion of KCl buffer, filtered through a cellulose acetate filter (pore size of ϕ0.20 μm; Advantec Toyo, Tokyo, Japan), was added to the two compartments of the assembled Teflon^®^ chamber. The buffer level in both compartments was initially set around the bottom edge of the lowest wells. A 80 μL portion of a lipid solution (10 mg/mL Egg-PC:Egg-PE:Chol = 7:1:2 (*w*/*w*) in chloroform:*n-*hexane = 1:1 (*v*/*v*)) was then carefully spread on the KCl buffer surface. After ~15 min, during which the solvent evaporated, the buffer level was gradually raised over the apertures to form BLMs, as illustrated in Figure 2. The rate of buffer injection was maintained at 6 mL/min using a syringe pump (KDS-260, KD Scientific, Holliston, MA, USA). This folding process was monitored by recording the electrical current between two Ag/AgCl electrodes inserted into the KCl buffer of the two compartments.

### 2.5. Parallel Recordings of hERG Channel Activities

**Figure 3:**
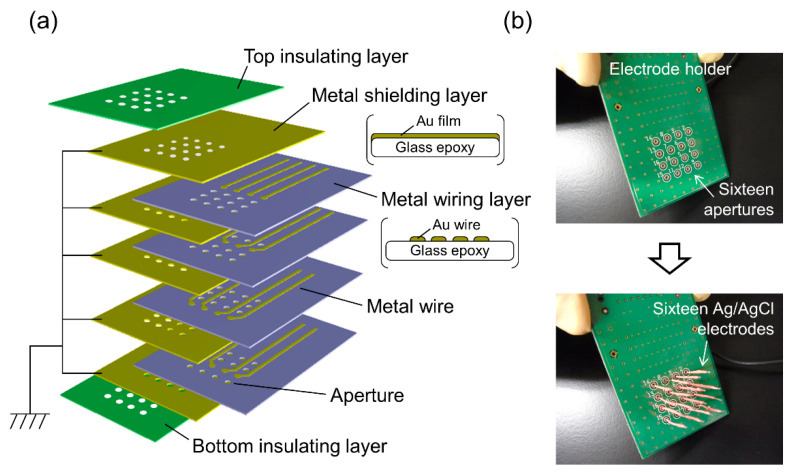
(**a**) Schematic drawing of the electrode holder. (**b**) Photographs of the electrode holder before and after inserting 16 Ag/AgCl electrodes.

After BLM formation, the compartment next to chip holder B was detached. Due to the small volume of the wells in chip holder B, the KCl buffer was retained in the wells after the detachment of the compartment (Figure 2). Sixteen Ag/AgCl electrodes were placed on a custom-built electrode holder, which was connected to a 16-channel patch clamp amplifier (Triton^+^, Tecella, CA, USA). The electrode holder had four wiring layers and metal shielding layers were sandwiched between the wiring layers, as illustrated in Figure 3a. The shielding layers were connected to the ground. The Ag/AgCl electrodes, as shown in Figure 3b, were inserted into the KCl buffer in the wells of chip holder B, which was called the *cis* side. Another Ag/AgCl electrode placed in the other compartment, which was called the *trans* side, was used as a common ground electrode (Figure 2). Transmembrane voltage was applied between the common ground electrode and Ag/AgCl electrode placed in each well. For incorporation of the hERG channels into the BLMs, 3 μL portions of proteoliposomes containing the hERG channels were added by pipetting to the respective wells. The proteoliposomes were added to only the *cis* side. After ~10 min, during which the proteoliposomes incorporated into the BLMs, current recordings were performed with a Triton^+^ 16-channel patch clamp amplifier, which was placed in a Faraday cage. The signals were recorded with a 1 kHz low-pass filter at a sampling frequency of 20 kHz. Additionally, single-channel currents were filtered offline at a cutoff frequency of 0.7 kHz [44]. Capacitance was measured by applying ramp voltage pulses of 1 V/s at a holding potential of 0 mV. The total capacitance (the sum of BLM and device capacitance) was 65 ± 6 pF (*n* = 105), and the capacitance of the electrode holder was ~38 pF. Typically, approximately an hour was required to record the ion-channel currents after the Si chips were mounted on chip holder A.

## 3. Results and Discussion

### 3.1. BLM Formation

**Figure 4:**
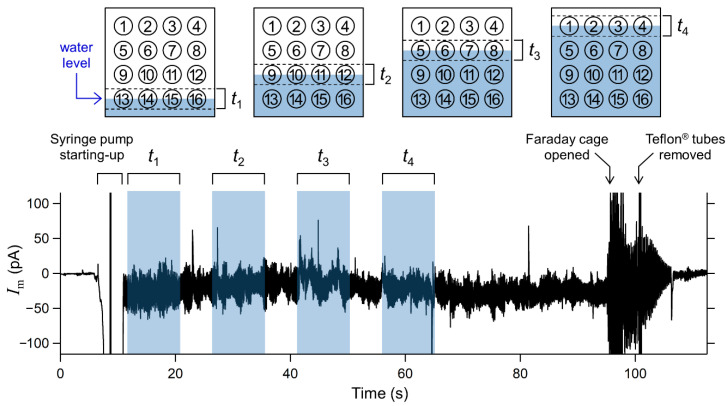
Electrical current flowing between two Ag/AgCl electrodes inserted in the two compartments, as shown in Figure 2. Applied voltage was 0 mV. Shaded regions represent expected times when buffer levels were gradually passing each row of the four wells. Schematic diagrams of the relationship between the buffer levels and the position of the wells corresponding to the respective shaded times are also shown.

In the present microarray system, all solvent-free BLMs were designed to be formed simultaneously by raising the buffer levels in the two compartments surrounding the micro-apertures. The two lipid monolayers spread at the air/buffer interface were folded up in the apertures to form BLMs. A current flowing between two Ag/AgCl electrodes inserted into the compartments was recorded when the KCl buffer was gradually added into the two compartments through Teflon^®^ tubes, which were connected to a syringe pump (Figure 4). Shaded regions represent the expected times when buffer levels gradually pass through each row of four wells of ϕ3 mm. The duration corresponding to each shaded region was ~9 s. Interestingly, the observed current exhibited significant noise when the buffer level reached the bottom edge of the wells, with the exception of the lowest four wells, for which the initial buffer level almost reached their bottom edges. When the compartments were filled with the KCl buffer and the Teflon^®^ tubes were removed from the chamber, the current level returned to zero, showing no leakage current across all the micro-apertures that were immersed in the KCl buffer. After detaching one of the compartments from the chamber, 11‒16 BLMs were usually formed with a membrane resistance higher than 1 GΩ. The average number of BLMs simultaneously formed was 13.1, across eight trials. The overall success probability of BLM formation was 82% (*n* = 128; 16 sites × 8 trials). Although most of the reported BLM arrays combined with electric recordings did not describe the formation probability of BLMs, only limited groups including us reported the formation probability (50‒61%) for arrays of BLMs based on non-volatile organic solvents [35,40]. Considering that the use of an organic solvent has been a common approach for the stable formation of BLMs [25], the observed superior formation probability of solvent-free BLMs to solvent-containing BLMs is probably due to the well-tuned tapered edge structure around the micro-apertures [45]. Thus, the folded BLMs can be formed simultaneously, and this process can be monitored by recording the total transmembrane current across all the Si chips.

**Figure 5:**
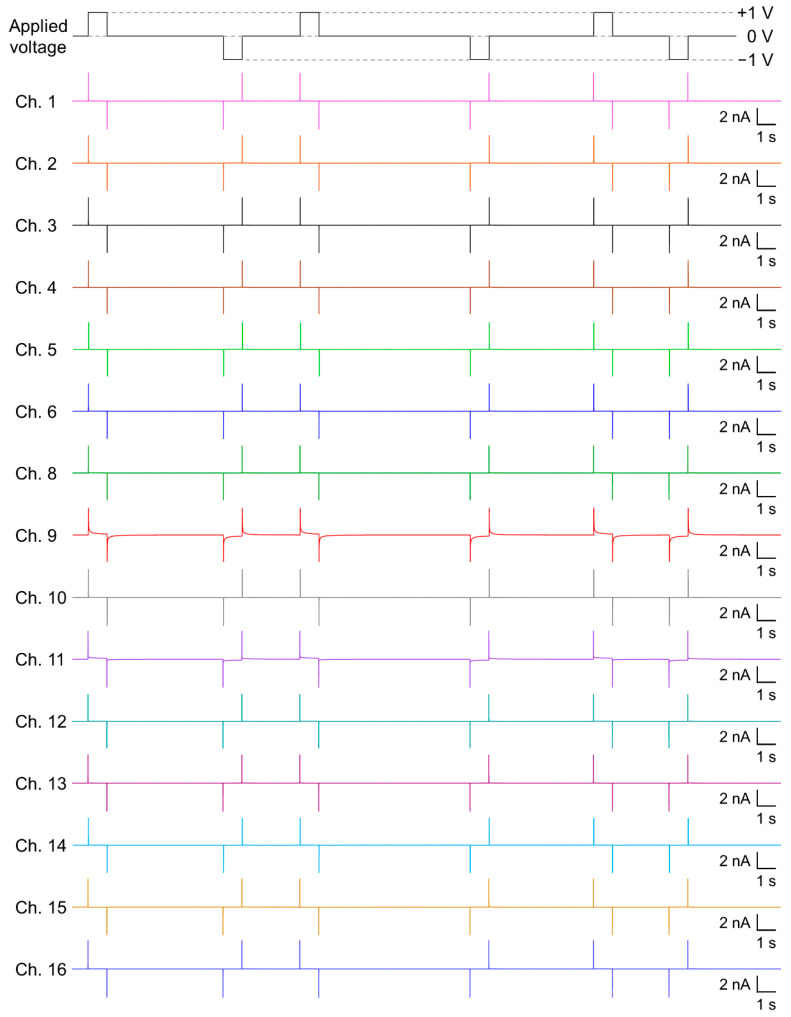
An example of simultaneously recorded transmembrane currents from the 15 BLMs, when the applied voltage was repeatedly switched from 0 to +1 V, from +1 to 0 V, from 0 to −1 V, and from −1 to 0 V.

Once the BLMs were formed on the multiple Si chips, the next step was to divide one compartment into individual wells for multiarray measurements. This step required detachment of the compartment filled with the KCl buffer and plugging off/on the Ag/AgCl electrodes, which may induce substantial mechanical and electric shocks to the solvent-free BLMs. However, owing to the tapered-edge structure of the micro-apertures, BLMs formed in the apertures were stable enough to tolerate the mechanical and electric shocks, leading to a high yield of BLM microarrays (vide supra). When voltage pulses of ±1 V were repeatedly applied to simultaneously formed 15 BLMs between a common ground electrode and individual electrodes placed in respective wells, all of the BLMs tolerated the pulse stimuli (Figure 5). This voltage magnitude was significantly higher than that of the breakdown voltage (0.2–0.6 V) reported for BLMs suspended in plastic and Si_3_N_4_ septa [23,46,47]. In addition, the breakdown voltage of BLMs formed in CPDS-modified glass nano- and micro-pores has been reported to be 0.4–0.8 V [20,21,22]. Considering that the surface of the present Si chips was also modified with CPDS, the improved stability to high voltages was probably due to the tapered-edge shape of the micro-aperture. The tapered aperture appeared suitable for reducing possible perturbation in the topology of the BLMs around the point of contact with the aperture edge. In addition, such high voltage stability is useful for enhancing the sensitivity of BLM systems [48,49,50] and detecting rare gating events of membrane proteins [50], which expands the potential of BLM systems.

### 3.2. Parallel Recordings of hERG Channel Activities in the Multiple BLMs

Next, in order to examine the potential of the BLM microarray system as a drug-screening platform, parallel recordings of ion-channel activities across multiple BLMs were investigated using the cardiac hERG channel as a representative example. The hERG channel is a voltage-dependent potassium channel that plays a crucial role in the electrical activity of the human heart, regulating repolarization during action potentials in cardiomyocytes [51,52]. This channel has attracted intense attention because diverse drugs have been found to block hERG channels, leading to unintentional side effects such as fatal arrhythmias [51,52]. A number of drugs, including antihistamines, gastric prokinetics, and antibiotics, have been withdrawn from the market due to their severe side effects on the hERG channel [6,51]. Regulatory agencies now require that the potential risks that drug candidates unintentionally inhibit hERG channel activities must be assessed before clinical trials are performed [6,51]. Therefore, it is critical to construct high-throughput systems for screening drug side effects on hERG channels.

**Figure 6:**
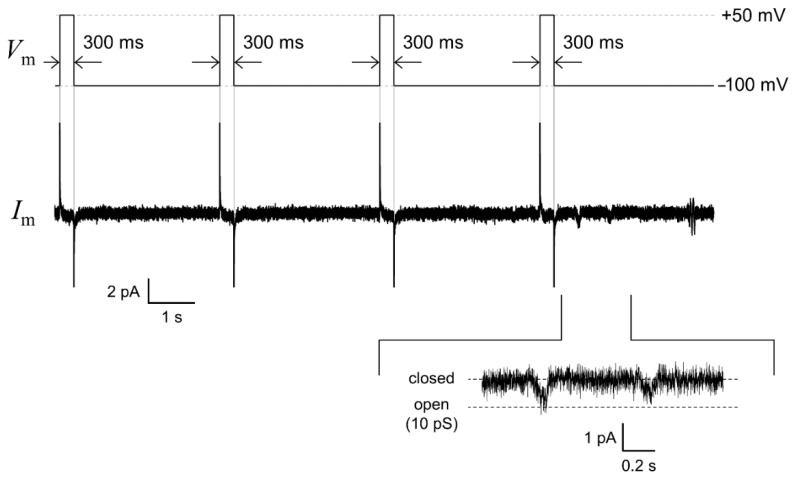
An example trace of the human *ether-a-go-go-*related gene (hERG) single-channel current (middle) recorded by applying a repetitive voltage-step protocol: −100 mV for 3.1 s after a 300 ms prepulse of +50 mV (**upper**). The enlarged current waveform is also displayed (**bottom**). The signals were recorded with a 1 kHz low-pass filter at a sampling frequency of 20 kHz. The current trace was filtered offline at a cutoff frequency of 0.7 kHz.

**Figure 7:**
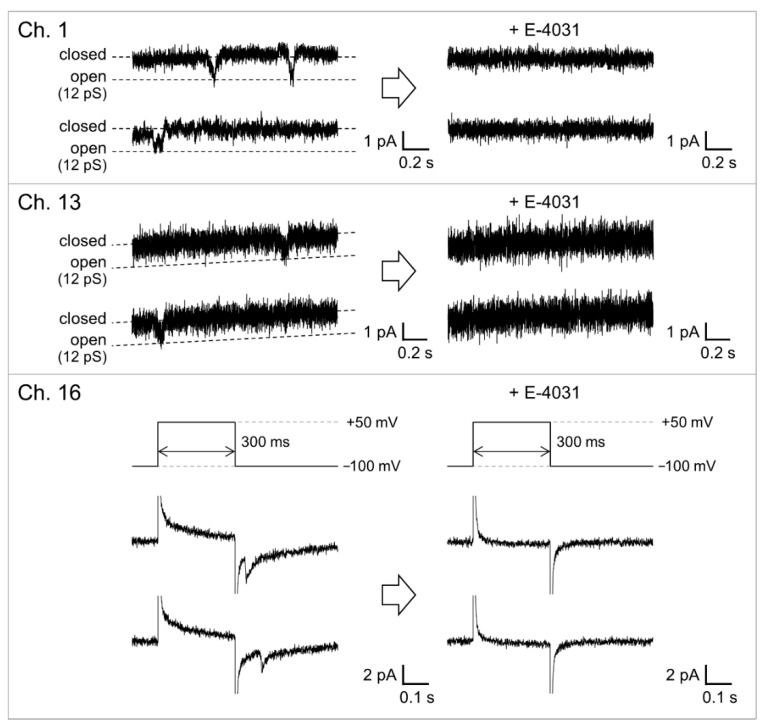
Parallel recordings of the hERG channel activities before and after the addition of E-4031. The voltage protocol shown in Figure 6 was used to elicit channel activities. For Ch. 1 and 13, current traces during the applied voltage kept at −100 mV are shown. For the current trace shown in Ch. 16, the applied voltage was switched from −100 to +50 mV and then switched back to −100 mV. E-4031 was added to the common side solution to give a final concentration of 10 μM. The signals were recorded with a 1 kHz low-pass filter at a sampling frequency of 20 kHz. The currents were filtered offline at a cutoff frequency of 0.7 kHz.

The hERG channels were incorporated into the solvent-free BLM microarray by adding proteoliposomes containing the hERG channels. The volume of the well was designed to be 40 μL to reduce the amount of hERG proteoliposomes, which made it possible to add the proteoliposome samples into multiple wells. Since the hERG channels are known to activate slowly and inactivate rapidly [51,53], we used a repetitive voltage pulse protocol to increase the probability of observing channel opening events. As shown in Figure 6, channel activities with a single-channel conductance of ~10 pS were observed in the fourth cycle of the voltage protocol. This conductance level was similar to that reported for the hERG channels in a BLM reconstitution system (10–14 pS) [30,54,55] and the hERG channels expressed in Xenopus oocytes [44]. Figure 7 shows examples of the hERG channel currents recorded from multiple wells with numbers 1, 13, and 16. Current responses, corresponding to single-channel activities, were observed at an applied voltage of −100 mV after a short prepulse to +50 mV (wells #1 and #13). In addition to these events, we frequently observed transient decaying current responses immediately after the voltage was switched from +50 to −100 mV, as shown in the current trace obtained from well #16. Similar decay currents were also reported for the ensemble-averaged hERG currents obtained with the patch-clamping method [44,56]. These channel activities were completely blocked when E-4031, a specific blocker [42,43], was added to the common side solution, where the ground Ag/AgCl electrode was inserted. It was demonstrated that hERG channel proteins were functionally incorporated into the BLMs. Thus, the hERG channel activities were recorded in parallel in multiple BLM systems. However, these channel activities were only observed in ~38% of BLMs, probably due to the low efficiency of channel incorporation through the fusion between BLMs and proteoliposomes. Previous reports on solvent-free BLM array platforms indicated an incorporation probability of ≥50% for α-hemolysin, which can be spontaneously integrated into BLMs by simply adding an α-hemolysin solution [9]. Therefore, the lower incorporation probability was probably due to the low efficiency of proteoliposome fusion. We previously reported that the application of centrifugal force to BLMs after the addition of proteoliposomes significantly increased the fusion efficiency and consequently the incorporation efficiency [41]. Further improvements in recording efficiency will be necessary, which can be achieved through the combination of the BLM microarray and centrifugal system for the realization of high-throughput drug-screening systems for ion-channel proteins.

## 4. Conclusions

In this paper, we presented a 16-site BLM microarray system based on microfabricated Si chips for simultaneous recordings of ion-channel activities. We succeeded in the simultaneous formation of solvent-free BLMs over 16 micro-apertures fabricated in Si chips. The probability of BLM formation reached 82% without the use of a non-volatile organic solvent. Parallel recordings of the hERG channel activities in the multiple BLMs were demonstrated, showing the potential of the present BLM system as a high-throughput screening platform for ion-channel proteins. The BLM reconstitution system is highly compatible with recently advanced cell-free expression systems [57,58], where target ion-channel proteins can be synthesized in vitro from protein-encoding DNA. The realization of high-throughput BLM microarray combined with cell-free expression systems will significantly increase the applicability of BLM reconstitution systems to various ion-channel genotypes. Considering that a relationship has been implied between the hERG channel genotypes and drug-induced arrhythmia [59], integration of the present BLM microarray with various hERG genotypes will provide a novel and efficient screening platform for assessing the potential risks of side effects of drugs acting on respective hERG channel genotypes.
